# Low Temperatures

**Published:** 1874-11

**Authors:** 


The Lowest Temperature Attainable.—Solidified carbonic acid gas dissolved in ether reduces the temperature to 140° below zero, Fahr. By evaporating this mixture in vacuo, the temperature falls to 166°. Solid carbonic acid mixed with solid nitrous oxyd and ether reduces it to 200°. By adding bi-sulphide of carbon to this mixture and evaporating in vacuo the temperature falls 20° lower, or to 220°, which is the greatest degree of cold yet attained.—*Pacific Medical and Surgical Journal.*

Removal of a Fragment of Iron from the Vitreous Humor by a Magnet.—*The Practitioner* for July contains a very interesting case of this kind, reported by Dr. McKeown, of Belfast, Ireland. A lad while hammering iron, received a fragment in the eye, which penetrated through the cornea and lodged deeply in the vitreous near the retina. Dr. McKeown made an incision in the sclerotic near the cornea and parallel with its edge, two and a half lines in length, and introduced a bar magnet, flat and tapering to a point, with which he penetrated the eye till its extremity reached the foreign body. The iron immediately attached itself to the magnet and on the third trial was completely extracted. The flat and tapering form of the bar is regarded by the Doctor as important on account of the plugging up the passage so as to prevent the escape of the humor during its introduction.

